# Identification and Validation of New Stable QTLs for Grain Weight and Size by Multiple Mapping Models in Common Wheat

**DOI:** 10.3389/fgene.2020.584859

**Published:** 2020-11-11

**Authors:** Jiajia Cao, Yaoyao Shang, Dongmei Xu, Kangle Xu, Xinran Cheng, Xu Pan, Xue Liu, Mingli Liu, Chang Gao, Shengnan Yan, Hui Yao, Wei Gao, Jie Lu, Haiping Zhang, Cheng Chang, Xianchun Xia, Shihe Xiao, Chuanxi Ma

**Affiliations:** ^1^KeyLaboratory of Wheat Biology and Genetic Improvement on Southern Yellow and Huai River Valley, Ministry of Agriculture and Rural Affairs, College of Agronomy, Anhui Agricultural University, Hefei, China; ^2^Institute of Crop Sciences, National Wheat Improvement Center, Chinese Academy of Agricultural Sciences, Beijing, China

**Keywords:** common wheat, grain weight/size, QTL, SLAF-seq, GWAS, SNP

## Abstract

Improvement of grain weight and size is an important objective for high-yield wheat breeding. In this study, 174 recombinant inbred lines (RILs) derived from the cross between Jing 411 and Hongmangchun 21 were used to construct a high-density genetic map by specific locus amplified fragment sequencing (SLAF-seq). Three mapping methods, including inclusive composite interval mapping (ICIM), genome-wide composite interval mapping (GCIM), and a mixed linear model performed with forward–backward stepwise (NWIM), were used to identify QTLs for thousand grain weight (TGW), grain width (GW), and grain length (GL). In total, we identified 30, 15, and 18 putative QTLs for TGW, GW, and GL that explain 1.1–33.9%, 3.1%–34.2%, and 1.7%–22.8% of the phenotypic variances, respectively. Among these, 19 (63.3%) QTLs for TGW, 10 (66.7%) for GW, and 7 (38.9%) for GL were consistent with those identified by genome-wide association analysis in 192 wheat varieties. Five new stable QTLs, including 3 for TGW (*Qtgw.ahau-1B.1*, *Qtgw.ahau-4B.1*, and *Qtgw.ahau-4B.2*) and 2 for GL (*Qgl.ahau-2A.1* and *Qgl.ahau-7A.2*), were detected by the three aforementioned mapping methods across environments. Subsequently, five cleaved amplified polymorphic sequence (CAPS) markers corresponding to these QTLs were developed and validated in 180 Chinese mini-core wheat accessions. In addition, 19 potential candidate genes for *Qtgw.ahau-4B.2* in a 0.31-Mb physical interval were further annotated, of which *TraesCS4B02G376400* and *TraesCS4B02G376800* encode a plasma membrane H^+^-ATPase and a serine/threonine-protein kinase, respectively. These new QTLs and CAPS markers will be useful for further marker-assisted selection and map-based cloning of target genes.

## Introduction

Wheat (*Triticum aestivum* L.) is one of the most important cereal crops and provides approximately 20% of the dietary calories for humans worldwide. The demand for wheat is continually increasing due to rapid population growth (Organization of Food and Agriculture, 2009). Grain weight and size, including thousand grain weight (TGW), grain length (GL), and grain width (GW), are positively associated with wheat grain yield (Börner et al., 2002; Dholakia et al., 2003; Breseghello and Sorrells, 2007). Therefore, dissecting the genetic basis of these grain-related traits contributes to the improvement of wheat yield.

Numerous quantitative trait loci (QTLs) underlying grain weight and size were detected on almost all wheat chromosomes by linkage mapping and genome-wide association studies (GWAS) (Cui et al., 2014; Simmonds et al., 2014; Wu et al., 2015; Thorwarth et al., 2019). Several candidate genes for TGW, GW, and GL have been isolated in wheat. *TaCKX6-D1* encodes a cytokinin oxidase/dehydrogenase (CKX2) associated with grain number and grain weight (Ashikari et al., 2005; Li et al., 2011, 2013). *TaGW2-6A* encodes a RING-type E3 ubiquitin ligase and functions as a negative regulator of GW (Su et al., 2012; Yang et al., 2012; Vandana et al., 2015). *TaCwi-A1* encodes a cell wall invertase for carbon partitioning during early grain filling (Wang et al., 2008; Ma et al., 2012). *TaSAP-A1* belongs to a gene family of stress-associated proteins significantly related to TGW, number of grains per spike, and spike length (Chang et al., 2013). *TaGS1a* encodes a cytosolic glutamine synthetase underlying grain size (Ying et al., 2013). *TaGS-D1* is associated with TGW and GL (Zhang et al., 2014). *TaSus1* and *TaSus2* encode two isoforms of sucrose synthase (Jiang et al., 2011; Hou et al., 2014). *TaGS5-3A* regulates grain size (Ma L. et al., 2015). *6-SFT-A2* is involved in fructan biosynthesis and significantly associated with TGW under rainfed conditions (Yue et al., 2015). *TaSnRK2.3*, *TaSnRK2.9*, and *TaSnRK2.10*, three members of the SnRK2 family, are associated with TGW (Miao et al., 2017; Zhang Z. et al., 2017; Rehman et al., 2019). More recently, Cheng et al. (2020) isolated the *Tasg-D1* gene, which encodes serine/threonine protein kinase glycogen synthase kinase 3 (STKc_GSK3), leading to formation of round grains in Indian dwarf wheat (*Triticum sphaerococcum* Perc.). *TaSDIR1-4A*, which encodes a RING-type E3 ubiquitin ligase is associated with TGW in well-watered and heat-stress environments (Wang J. et al., 2020). The physical interaction of the proteins encoded by *TaDA1-A* and *TaGW2-6B* was significantly associated with grain size and weight (Liu H. et al., 2019; Liu J. et al., 2019). *TaGS3-7A*, a homologous gene of *OsGS3*, was shown to be associated with grain weight (Yang et al., 2019b). In addition, *TaTGW6* (Hu et al., 2016a), *Tabas1* (Zhu et al., 2016), *TaCKX4* (Chang et al., 2015), *TaFlo2* (Sajjad et al., 2017), *TaTGW-7A* (Hu et al., 2016b), *TaCYP78A3* (Ma M. et al., 2015), and *TaGL3* (Yang et al., 2019a) are also significantly associated with grain weight or size. All of the genes listed above are useful in genetic improvement of wheat yield. However, wheat grain weight and size are complex traits controlled by multiple genes. Identification and validation of more QTLs for grain-related traits will not only promote our understanding of the genetic basis of the two traits, but also accelerate the process of pyramiding favorable alleles in high-yield wheat breeding.

In the past decades, QTL mapping and GWAS were performed based on randomly amplified polymorphic DNA (RAPDs) (Qu and Hancock, 1997), restriction fragment length polymorphisms (RFLPs) (Keim et al., 1990), amplified fragment length polymorphisms (AFLPs) (Vos et al., 1995; Chagne et al., 2002), simple sequence repeats (SSRs) (Torada et al., 2005), and sequence-tagged site (STS) markers (Talbert et al., 1994). However, the number of these markers is limited, and this affects the accuracy and efficiency of QTL mapping and GWAS.

In recent years, several approaches for high-throughput molecular markers supported by next-generation sequencing technologies and SNP chips have developed rapidly, including DNA sequencing (RAD-seq) (Baird et al., 2008), genotyping-by-sequencing (GBS-seq) (Elshire et al., 2011), restriction site-associated specific locus amplified fragment sequencing (SLAF-seq) (Sun et al., 2013), and wheat 90K (Wang et al., 2014), 55K, and 660K SNP arrays (designed by the Chinese Academy of Agricultural Sciences) (Cui et al., 2017; Huang et al., 2019). These high-throughput genotyping methods obviously accelerate the identification of novel loci and candidate genes underlying wheat yield–related traits. Cui et al. (2017) constructed a genetic map with 4959 bin markers using a wheat 660K SNP array in a recombinant inbred line (RIL) population derived from Kenong 9204 × Jing 411 and detected a major QTL for kernel number per spike on chromosome 4A. Fiedler et al. (2017) identified a QTL for test weight on chromosome 1B by GBS-seq of 1184 lines collected from the North Dakota State University durum wheat breeding program. Wang F. et al. (2020) constructed a high-density genetic map (HDGM), including 3556 SLAF-based SNP and SSR markers, and detected 37 QTLs related to yield traits in an RIL population derived from cotton cultivars LMY 22 (high-yield) and LY 343 (superior fiber quality). Notably, SLAF-seq has been widely applied to construct a HDGM for QTL mapping as well as narrowing target regions in several crops, such as maize (Liu et al., 2016; Li et al., 2018), rice (Yang et al., 2017; Quan et al., 2018), soybeans (Li et al., 2017; Han et al., 2019), and peppers (Guo et al., 2017; Zhang et al., 2019). However, few HDGMs have been constructed by SLAF-seq for common wheat.

The aims of this study were to (1) identify a large number of SNP markers to construct a HDGM by SLAF-seq in an RIL population derived from a cross between high-TGW cultivar Jing 411 and low-TGW landrace Hongmangchun 21, (2) identify QTLs for grain weight and size using SNP markers, and (3) design cleaved amplified polymorphism sequences (CAPS) markers closely linked to new stable QTLs for grain weight and size and validate them in 192 wheat varieties and 180 Chinese mini-core wheat accessions.

## Materials and Methods

### Plant Materials

A RIL population (174 lines) derived from a cross between Jing 411 (average TGW: 41.94 g) and Hongmangchun 21 (average TGW: 19.98 g) (designated JH-RILs) was used for QTL mapping; it was planted during the 2005–2006, 2006–2007, 2007–2008, 2009–2010, 2010–2011, 2011–2012, 2013–2014, 2014–2015, 2015–2016, 2016–2017, and 2017–2018 cropping seasons. One hundred ninety-two wheat varieties (WVs) grown in the 2015–2016, 2016–2017, and 2017–2018 cropping seasons, containing 159 cultivars, 11 advanced breeding lines, and 22 landraces, were chosen for a GWAS panel to validate QTLs identified by linkage analysis. The origin of these varieties has been described in detail by Zhu et al. (2019). One hundred eighty Chinese mini-core collection accessions (CMCCs) planted in the 2014–2015, 2015–2016, and 2016–2017 cropping seasons comprising 74 cultivars, 11 advanced breeding lines, and 95 landraces were used to validate new stable loci for grain weight and size.

The above three populations were planted at the experimental station of Anhui Agricultural University in Hefei (31°58′N, 117°240′E), Anhui Province, China. Field trials were conducted in 2-m-long plots, each consisting of double rows spaced 0.25 m apart in randomized complete blocks with two replications.

### TGW, GW, and GL Tests

Sixty spikes per plot were harvested and threshed at physiological maturity, which is characterized by yellow color on the whole plant including leaves, stems, and spikes (Kulwal et al., 2005). Three hundred threshed seeds were used to measure TGW, GL, and GW in triplicate using the SC-G wheat grain appearance quality image analysis system (Hangzhou WSeen Detection Technology Co., Ltd, Hangzhou, China) (Yin et al., 2015).

For JH-RILs, TGW was measured during the 2005–2006, 2006–2007, 2007–2008, 2009–2010, 2010–2011, 2011–2012, 2013–2014, 2014–2015, 2015–2016, 2016–2017, and 2017–2018 cropping seasons, and these measurements were designated 2006TGW-JH, 2007TGW-JH, 2008TGW-JH, 2010TGW-JH, 2011TGW-JH, 2012TGW-JH, 2014TGW-JH, 2015TGW-JH, 2016TGW-JH, 2017TGW-JH, and 2018TGW-JH, respectively. GW and GL were measured in part seasons, and these measurements were designated 2014GW-JH, 2015GW-JH, 2016GW-JH, 2017GW-JH, 2018GW-JH, 2014GL-JH, 2015GL-JH, 2016GL-JH, 2017GL-JH, and 2018GL-JH, respectively.

For 192 WVs, TGW was evaluated during the 2015–2016, 2016–2017, and 2017–2018 cropping seasons, designated 2016TGW-192, 2017TGW-192, and 2018TGW-192, respectively; GW and GL were also measured, designated 2016GW-192, 2017GW-192, 2018GW-192, 2016GL-192, 2017GL-192, and 2018GL-192, respectively.

For 180 CMCCs, TGW was evaluated during the 2014–2015, 2015–2016, and 2016–2017 cropping seasons, designated 2015TGW-CMCC, 2016TGW-CMCC, and 2017TGW-CMCC, respectively; GW and GL were measured and designated 2015GW-CMCC, 2016GW-CMCC, 2017GW-CMCC, 2015GL-CMCC, 2016GL-CMCC, and 2017GL-CMCC, respectively.

### Statistical Analysis

Pearson’s correlation analysis and Mann–Whitney *U*-tests were performed by the software SPSS 20.0 (IBM Corporation, Armonk, NY, United States).

### Genomic DNA Isolation

Dry seeds were collected to extract genomic DNA using the SDS method (Kang et al., 1998). DNA quality was tested by ND5000 spectrophotometer (NanoDrop, Wilmington, DE, United States) and in 1% agarose gels.

### Construction of SLAF Library

The JH-RILs were genotyped by a modified SLAF-seq strategy to develop genome-wide SNP markers (Sun et al., 2013). The Chinese Spring reference genome IWGSC RefSeq v1.0^1^ was utilized to simulate *in silico* the number of markers digested by different restriction enzymes and design a pilot SLAF experiment (Sun et al., 2013). A restriction enzyme (*Rsa*I, New England Biolabs, United States) was used to digest the genomic DNA. The fragments ranging from 464 to 484 bp were selected and purified after agarose gel electrophoresis for 100-bp paired-end sequencing. To assess the accuracy of the experiment for SLAF library construction, *Oryza sativa* L. *japonica* was chosen as a control in comparison to mapping populations with the same experimental treatment and sequencing.

### SLAF-Seq Data Analysis and Genotyping

In order to ensure the accuracy of data analysis, quality control for the raw sequencing data were performed according to the following criteria: (i) reads containing adaptor sequences were filtered out, (ii) reads with unknown bases exceeding 10% in length were filtered out, (iii) reads unaligned to the wheat reference genome IWGSC RefSeq v1.0 (see footnote 1) were filtered out, and (iv) reads with a single end aligned to the wheat reference genome IWGSC RefSeq v1.0 were also filtered out because of the inaccurate physical position.

SLAF paired-end mapped reads were clustered based on sequence similarity identified by alignments to the wheat reference genome IWGSC RefSeq v1.0 with BWA software (Chong et al., 2003). SNPs in all SLAF loci were identified between parents by the Genome Analysis Toolkit (McKenna et al., 2010)^2^. SLAFs with 8 or more SNPs, which are considered as high-frequency variable regions of wheat, were filtered out, and this influenced the accuracy of following steps. Minor allele frequency evaluation was used to define alleles in each SLAF. SLAF markers were generated following Sun et al. (2013). Polymorphic markers were selected by genotyping the parents and then classified into eight segregation types (ab × cd, ef × eg, hk × hk, lm × ll, nn × np, aa × bb, ab × cc, and cc × ab). For the RIL population, SLAF markers in the segregation pattern of aa × bb were selected. The quality of SLAF markers used to build genetic maps was controlled by the following criteria: (i) average sequence depths must be >6-fold in parents, (ii) markers with more than 25% missing data were filtered out, (iii) markers with significant segregation distortion (*P* < 0.001) were rejected, and (iv) markers were required to have a logarithm of odds (MLOD) exceeding 3.

### Bulked Segregant Analysis of JH-RILs by 660K SNP Arrays

Two high-TGW bulks (HB1 and HB2) and two low-TGW bulks (LB1 and LB2) were made by the same amount of DNA from extremely high and low TGW lines (5 lines per bulk) of JH-RILs based on phenotypic values. The above four bulks and two parents were genotyped using the wheat 660K SNP array by the CapitalBio Corporation (Beijing, China). SNP genotyping and clustering were performed by Genome Studio Polyploid Clustering v1.0^3^. Consistent SNPs without missing data in HB1, HB2, and Jing 411 were classified as group I, and consistent SNPs without missing data in LB1, LB2, and Hongmangchun 21 were classified as group II. Different SNPs between groups I and II were selected for subsequent analysis and development of PCR-based molecular markers. Physical positions of these SNPs were searched by sequence BLAST with IWGSC RefSeq v1.0 (see footnote 1).

### Development and Genotyping of PCR-Based Molecular Markers

Seventy-four SNPs with polymorphisms between groups I and II were converted to CAPS markers by Primer Premier 5.0^4^ and used to genotype JH-RILs to construct genetic maps (Supplementary Table 1). Representative CAPS markers closely linked with new stable QTLs were used to genotype 180 CMCCs. A PCR mixture with a total volume of 10 μL comprised 1.0 μL of 10 × PCR buffer, 200 μM dNTPs, 4 pmol of each primer, 0.5 U *Taq* DNA polymerase, and 50–100 ng template DNA. The reaction was performed in a C1000 Thermal Cycler (Bio-Rad, United States) with the following program: denaturation at 94°C for 5 min followed by 40 cycles at 94°C for 30 s, touchdown starting at 62°C for 30 s (decreasing 0.3°C per cycle), and 72°C for 30 s that were followed by a final extension at 72°C for 8 min. The PCR products were digested with the corresponding restriction enzymes (Supplementary Table 1^5^) for 5 h and separated on 2.5% agarose gels.

Eleven functional markers specific to *TaSdr-2A* (Zhang Y. et al., 2017), *TaSus-2A* (Hou et al., 2014), *Tamyb10-3A* (Himi et al., 2011), *TaVp1B3-3B* (Yang Y. et al., 2007), *TaVp1-b2* (3B) (Chang et al., 2010), *Tamyb10-3D* (Himi et al., 2011), *TaTGW6-4A* (Hu et al., 2016a), *TaMKK3-4A* (Torada et al., 2016), *TaGW2-6A* (Su et al., 2012), *TaGASR7-A1* (7A) (Dong et al., 2014), and *TaPTF1-7B* (Zhu, 2016) were used for QTL analysis in JH-RILs in combination with SLAF and CAPS markers. PCR conditions and gel electrophoresis for functional markers were performed following the above reported studies (Supplementary Table 2).

### Genetic Linkage Map Construction and QTL Mapping

SLAF markers distributed on 21 wheat chromosomes were selected by mapping using IWGSC RefSeq v1.0 (see footnote 1). To ensure the accuracy of genetic linkage maps, the MLOD scores between markers were estimated, and markers with MLOD scores less than 5 were removed. Then, SLAF, CAPS, and gene-specific markers were collectively used to construct a high-density genetic bin map (designated SLAF-map) without redundant markers by IciMapping v4.1 using the Kosambi mapping function in JH-RILs.

The inclusive composite interval mapping (ICIM) program of QTL IciMapping v4.1 (Li et al., 2007)^6^ was used to detect QTLs for TGW, GL, and GW in JH-RILs with a walk speed of 1 cM and a window size of 10 cM. The genome-wide composite interval mapping (GCIM) of QTL.gCIM.GUI v1.0 (Wen et al., 2019)^7^ in R version 3.6.0 software was applied to identify QTLs with a random model and a walk speed of 1 cM. For these two methods, an LOD score of 2.5 was used for claiming the presence of QTLs. The QTLnetwork v2.0 software (Yang J. et al., 2007; Yang et al., 2008), which is based on a mixed linear model (MLM), was used for forward–backward stepwise (NWIM) analysis with a threshold of *P* = 0.05 to select cofactors, multiple linear regression with a 1 cM walk speed, and a window size set at 10 cM.

Adjacent QTLs with the same sign of additive effects satisfying at least one of the following criteria were defined as the same QTL: (1) positions of QTL peaks within 10 cM (Kumar et al., 2016) and (2) QTLs with overlapped confidence intervals (Cui et al., 2017). QTLs for TGW detected in more than five environments and those for GW or GL in more than three environments were defined as stable loci (Cui et al., 2014).

### Genotyping and Association Analysis of 192 WVs Using 90K SNP Array

The DNA samples of 192 WVs were genotyped by the Illumina iSelect 90K Infinium SNP array, including 81,587 SNPs (Wang et al., 2014), by the Beijing Compass Biotechnology Co., Ltd. Genotypic clusters for each SNP were determined by the Genome Studio version 2011.1 software (Illumina, see footnote 3). The physical positions of SNPs were obtained from IWGSC RefSeq v1.0 (see footnote 1). The SNPs with less than 20% missing data and a minor allele frequency exceeding 5% were used for association analysis.

Linkage disequilibrium and population structure were analyzed for 192 WVs following methods in our previous study (Zhu et al., 2019). The same K and Q matrix data were used in the present study to identify significant marker–trait associations (MTAs) for grain weight and size by the MLM. The MLM was performed by TASSEL 5.0 to detect MTAs at a significance level of *P* < 0.001 (Bradbury et al., 2007; Chen et al., 2016).

### Gene Annotation

Databases from the IWGSC gene annotation^8^ were used to perform gene function annotations by BLAST.

### Cloning of *TraesCS4B02G376400*

Primer pair PMA-1 was designed to amplify the partial sequence of *TraesCS4B02G376400* based on the putative sequence from IWGSC RefSeq v1.0 (see footnote 1) (Supplementary Table 3). PCR amplifications were performed in 10-μL volumes that included 0.25 μM of each primer, 0.25 mM dNTPs, 100 ng genomic DNA, 0.5 unit *Fastpfu* polymerase, and 1 μL of 10 × *Fastpfu* PCR buffer (Beijing TransGen Biotech, Beijing, China). The amplification program consisted of an initial denaturation at 94°C for 5 min followed by 36 cycles of denaturation at 94°C for 45 s, annealing at 52°C for 50 s, and extension at 72°C for 1 min that were followed by a final extension at 72°C for 12 min. Amplified PCR fragments were separated on 1.5% agarose gels. Target fragments were recovered and cloned into a Blunt-zero vector and transformed into T1 competent cells (Beijing TransGen Biotech, Beijing, China).

Sequencing was carried out on an ABI 3500 genetic analyzer (Applied Biosystems, Shanghai, China). Sequence alignments were performed using DNAMAN 6.0^9^.

### Development of CAPS Marker for *TraesCS4B02G376400*

The SNP distinguished by restriction enzyme *Sty*I found in *TraesCS4B02G376400* between Jing 411 and Hongmangchun 21 was converted to a CAPS marker PMA-2 by Primer Premier 5.0 (see footnote 4) and used to genotype 180 CMCCs (Supplementary Table 3). The PCR conditions for the marker PMA-2 were consistent with those for 74 CAPS markers mentioned above.

## Results

### Statistical Analysis of Phenotypic Data

Phenotypic data for TGW, GW, and GL exhibited extensive variations in JH-RILs and 192 WVs across environments. In JH-RILs, the ranges of TGW, GW, and GL were 13.71–68.28 g (mean 35.97 g), 2.25–4.38 mm (mean 3.01 mm), and 3.99–8.55 mm (mean 6.36 mm), respectively. In the 192 WVs, TGW, GW, and GL ranged from 19.58 to 54.21 g (mean 38.29 g), 1.21 to 4.68 mm (mean 3.01 mm), and 4.37 to 7.50 mm (mean 6.23 mm), respectively (Supplementary Table 4).

Highly significant correlations were observed among TGW, GW, and GL values in different environments. TGW showed positive correlations with GW and GL with correlation coefficients ranging from 0.39–0.97 (average 0.67) and 0.43–0.94 (average 0.76) in JH-RILs and from 0.28–0.84 (average 0.54) and 0.19–0.72 (average 0.47) in the 192 WVs (Supplementary Tables 5, 6).

### Construction of Genetic Linkage Maps

After SLAF library construction and high-throughput sequencing, 576.7 M clean reads were obtained with each read being 200 bp. The number of SLAF tags was 816,183 for Jing 411; 798,085 for Hongmangchun 21; and 462,529 for progenies. The average depths of the SLAF markers were 21.52 in Jing 411, 19.64 in Hongmangchun 21, and 4.16 in progenies. Based on the filtering criteria described above, 1529 SLAF bin markers containing 4820 high-quality polymorphic SLAF markers in combination with 74 CAPS and 11 functional markers were used for QTL mapping (Supplementary Figure 1). Finally, a high-density genetic bin map, including 1529 SLAF bin markers, 74 CAPS markers, and 11 gene-specific functional markers spanned 2014.43 cM in length with an average marker interval of 1.22 cM. The genetic distances of the A, B, and D genomes were 864.2 cM (608 markers), 900.0 cM (764 markers), and 250.2 cM (242 markers), respectively (Supplementary Figure 1 and Supplementary Table 7).

### QTLs for TGW, GW, and GL Identified by Linkage Analysis

**FIGURE 1:**
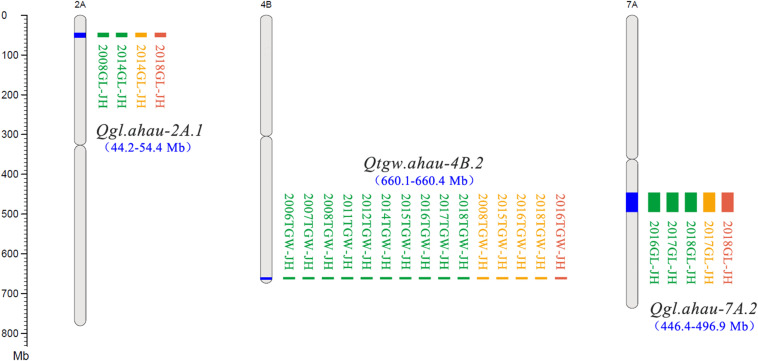
New stable QTLs for TGW and GL in the Jing 411/Hongmangchun 21 population. Uniform million bp (Mb) scale is shown on the left. Green, orange, and red blocks indicate QTLs detected by ICIM performed by QTL IciMapping v4.1, GCIM using QTL.gCIM.GUI v1.0, and composite interval mapping based on mixed linear model using forward–backward stepwise (NWIM) in QTLNetwork v2.0, respectively.

A total of 30 QTLs for TGW were identified using three mapping methods (ICIM, GCIM, and NWIM) in JH-RILs and were distributed on chromosomes 1B (3), 2A (3), 2B (2), 2D, 3A (3), 3B, 4A (2), 4B (2), 4D, 5A (3), 6A, 6B (2), 7A (2), 7B (2), and 7D (2). These QTLs explained 1.1–33.9% of the phenotypic variance, especially five QTLs (*Qtgw.ahau-2A.3*, *Qtgw.ahau-5A.1*, *Qtgw.ahau-7A.2*, *Qtgw.ahau-7B.1*, and *Qtgw.ahau-7B.2*) that had relatively high phenotypic variance explained (PVE) ranging from 10.3 to 16.5%. In addition, 13 QTLs for TGW were detected by two methods across environments and were distributed on chromosomes 2A (2), 3A, 3B, 4A, 4D, 5A (2), 7A (2), 7B (2), and 7D. Five QTLs, including *Qtgw.ahau-1B.1*, *Qtgw.ahau-2D*, *Qtgw.ahau-4B.1*, *Qtgw.ahau-4B.2*, and *Qtgw.ahau-6B.1*, were collectively detected by all three methods across environments. Particularly, *Qtgw.ahau-4B.2* was identified in 10 environments, explaining 11.7% of the phenotypic variances on average and, thus, was considered a stable and major QTL (Figure 1, Table 1, and Supplementary Table 8).

**TABLE 1:** QTLs for grain weight and size identified by all three mapping methods in the Jing 411/Hongmangchun 21 RIL population.

**Trait**	**QTL**	**Physical Interval (Mb)^b^**	**Representative Marker**	**No. of Environments**	**Method^c^**	**Peak Position (cM)**	**LOD or *P*-value^d^**	**PVE^e^ (%)**	**Additive Effect**	**References**
TGW	*Qtgw.ahau-1B.1*	648.1–652.1	1B-JHMfeI	5	ICIM	184.0–185.0	3.27–4.38	5.4–8.0	1.46–2.23	**Novel** for TGW;
					GCIM	184.8–185.4	2.74–5.42	3.5–6.3	1.85–2.17	Cui et al., 2014
					NWIM	187.2	0.00	19.8	2.37	(**KNS**)
	*Qtgw.ahau-2D*	543.5–561.9	2D-5137	2	ICIM	60.0–62.0	3.27–6.45	8.2–8.5	2.03	Liu et al., 2018
					GCIM	61.5	3.52	5.0	1.94	
					NWIM	61.3	0.00	30.4	3.34	
	*Qtgw.ahau-4B.1^a^*	540.3–542.9	4B-8621	5	ICIM	34.0–44.0	5.05–14.74	9.6–29.3	1.97–4.77	**Novel**
					GCIM	43.5	2.57–3.70	1.9–3.5	1.93–2.09	
					NWIM	35.0	0.00	17.8	4.13	
	*Qtgw.ahau-4B.2^a^*	660.1–660.4	4B-9051	10	ICIM	115.0–116.0	3.37–10.25	5.2–23.2	1.47–3.26	**Novel**
					GCIM	115.2–116.2	2.80–8.63	2.6–15.0	1.63–3.10	
					NWIM	115.2	0.00	33.9	5.72	
	*Qtgw.ahau-6B.1*	677.7–679.6	6B-9077	3	ICIM	73.0	4.22	8.2	1.80	Li et al., 2019
					GCIM	73.3	2.86	3.7	1.96	
					NWIM	73.4	0.00	18.1	3.82	
GW	*Qgw.ahau-2D.3^a^*	543.5–561.9	2D-5137	1	ICIM	62.0	8.36	14.0	0.09	Liu et al., 2018
					GCIM	60.5	4.98	14.7	0.10	
					NWIM	60.6	0.00	26.7	0.12	
	*Qgw.ahau-4B.3^a^*	660.11–660.13	4B-9051	2	ICIM	116.0	4.21–14.27	11.0–20.7	0.06–0.10	**Novel**
					GCIM	116.2	3.44–7.31	8.1–26.0	0.09–0.17	
					NWIM	115.2	0.00	34.2	0.17	
GL	*Qgl.ahau-2A.1^a^*	44.2–54.4	2A-CAPSmin	3	ICIM	105.0–107.0	6.54–8.96	13.8–15.5	0.21–0.25	**Novel**
					GCIM	103.8	6.42	10.4	0.22	
					NWIM	211.0	0.01	8.5	0.25	
	*Qgl.ahau-4B.1*	31.3–38.4	Marker13172883	2	ICIM	6.0	5.16–16.88	6.5–18.0	0.14–0.22	Garcia et al., 2019
					GCIM	6.3–7.2	4.75–3.68	2.1–5.3	0.12–0.20	
					NWIM	6.4	0.00	11.9	0.19	
	*Qgl.ahau-6B*	676.8–678.2	6B-6004	4	ICIM	80.0–86.0	3.11–5.79	4.8–5.8	0.12–0.14	Li et al., 2019
					GCIM	88.0	3.17	1.7	0.11	
					NWIM	91.8	0.01	7.7	0.22	
	*Qgl.ahau-7A.2^a^*	446.4–496.9	7A-3738	3	ICIM	123.0	3.75–15.88	9.7–22.8	0.17–0.38	**Novel** for GL;
					GCIM	122.8	5.59	6.3	0.22	Wang et al., 2017
					NWIM	63.2	0.02	9.5	0.20	(**KNS**)

*^*a*^The same loci detected by linkage analysis in the JH RIL population and association analysis in 192 wheat varieties. ^*b*^The physical positions of SNP markers based on wheat genome sequences from the International Wheat Genome Sequencing Consortium (IWGSC Ref Seq 1.0, http://www.wheatgenome.org/). ^*c*^ICIM indicates the inclusive composite interval mapping method performed by QTL IciMapping v4.1, GCIM indicates the genome-wide composite interval mapping method of QTL. gCIM.GUI v1.0, NWIM indicates composite interval mapping based on mixed linear model performed using forward–backward stepwise in QTLNetwork v2.0. ^*d*^LOD values from ICIM and GCIM > 2.5, *P-*values from NWIM < 0.05. ^*e*^PVE indicates phenotypic variance explained.*

Fifteen QTLs associated with GW were mapped on chromosomes 1B, 2D (3), 3B, 4A, 4B (3), 4D, 5A, 5B, 7A (2), and 7B using the aforementioned three mapping methods. Among them, 6 QTLs for GW on chromosomes 1B, 2D, 3B, 4A, 4B, and 4D were detected by two methods across environments. Notably, two QTLs (*Qgw.ahau-2D.3* and *Qgw.ahau-4B.3*), which explained 18.5 and 20.0% of the phenotypic variances on average, respectively, were jointly detected by all three methods (Table 1 and Supplementary Table 8).

Eighteen QTLs for GL were identified on chromosomes 1B (3), 2A (3), 2B, 2D, 3A, 4B (4), 6B, 7A (3), and 7B with three mapping methods. Of these, two QTLs, *Qgl.ahau-1B.1* and *Qgl.ahau-4B.3*, were identified by two methods in more than one environment. Three QTLs, including *Qgl.ahau-2A.1*, *Qgl.ahau-6B*, and *Qgl.ahau-7A.2*, were consistently detected by all three methods in several environments. Of these, *Qgl.ahau-2A.1* and *Qgl.ahau-7A.2* explained 12.1% and 11.9% of the phenotypic variances, respectively, and were considered as stable and major QTLs (Figure 1, Table 1, and Supplementary Table 8).

### Validation of QTLs for TGW, GW, and GL by GWAS

Association analysis was performed to validate QTLs for TGW, GW, and GL detected in JH-RILs using 192 WVs in three environments based on an MLM (Supplementary Figure 2). The results indicated that 19 (63.3%) of 30 QTLs for TGW, 10 (66.7%) of 15 for GW, and 7 (38.9%) of 18 for GL detected in JH-RILs were consistent with those identified by GWAS in 192 varieties. Among them, two reported QTLs for GW (*Qgw.ahau-2D.1*, 272.5–420.5 Mb; *Qgw.ahau-4D*, 226.4–409.4 Mb) identified in JH-RILs were closely linked with two stable MTAs (*D_GB5Y7FA01EHPZX_186*, 353.1 Mb on chromosome 2D; *IACX65*, 352.4 Mb on chromosome 4D) as detected by GWAS in 192 WVs across all environments. Four reported intervals for TGW harboring *Qtgw.ahau-5A.3* (559.3–596.6 Mb), *Qtgw.ahau-6B.2* (679.6–698.3 Mb), *Qtgw.ahau-7A.1* (99.7–200.5 Mb), *Qtgw.ahau-7D.1* with positive addictive effect (256.1–383.1 Mb)/*Qtgw.ahau-7D.2* with negative addictive effect (270.6–391.9 Mb) identified in JH-RILs were near to four MTAs (*Excalibur_c56952_411*, 569.8 Mb on chromosome 5A; *RAC875_c17347_312*, 694.2 Mb on chromosome 6B; *RAC875_c5986_3670*, 200.1 Mb on chromosome 7A; *RAC875_c45846_454*, 324.5 Mb on chromosome 7D) detected in 192 WVs, explained 12.6, 11.0, 10.7, and 12.5% of the phenotypic variances on average, respectively. Notably, three novel and major QTLs, including *Qtgw.ahau-4B.2* (660.1–660.4 Mb), *Qgl.ahau-2A.1* (44.2–54.4 Mb), and *Qgl.ahau-7A.2* (446.4–496.9 Mb), were adjacent to three MTAs (*RAC875_c48025_483*, 666.1 Mb on chromosome 4B; *Tdurum_contig55610_784*, 42.5 Mb on chromosome 2A; *Kukri_c5101_2636*, 498.9 Mb on chromosome 7A) based on their physical positions in 192 wheat varieties, respectively (Table 1 and Supplementary Table 9).

### Validation of New Stable QTLs in 180 CMCCs

Four CAPS markers (1B-JHMfeI for *Qtgw.ahau-1B.1*, 2A-CAPSmin for *Qgl.ahau-2A.1*, 4B-8621 for *Qtgw.ahau-4B.1*, and 7A-3738 for *Qgl.ahau-7A.2*) were further developed to validate the associations of four new stable QTLs with grain weight and size in 180 CMCCs (Table 1 and Supplementary Tables 1, 8). Allelic variations of the CAPS markers were detected, designated as *1B-JHMfeI-C*/*1B-JHMfeI-T*, *2A-CAPSmin-T*/*2A-CAPSmin-C*, *4B-8621-C*/*4B-8621-G*, and *7A-3738-G*/*7A-3738-C*, respectively (Supplementary Figure 3). Compared with the Jing 411 genotypes (*1B-JHMfeI-C*, *2A-CAPSmin-T*, *4B-8621-C*, and *7A-3738-G*), the Hongmangchun 21 genotypes (*1B-JHMfeI-T*, *2A-CAPSmin-C*, *4B-8621-G*, and *7A-3738-C*) were significantly associated with lower TGW, GW, and GL values across environments (*P* < 0.01 or *P* < 0.05) (Table 2).

**TABLE 2:** Association of allelic variations of four CAPS markers for four novel QTLs with TGW, GW, and GL in 180 CMCC.

	**2015TGW-CMCC**	**2016TGW-CMCC**	**2017TGW-CMCC**	**2016GW-CMCC**	**2017GW-CMCC**	**2016GL-CMCC**	**2017GL-CMCC**
1B-JHMfeI-C^*a*^ Mean ± SD	38.46 ± 8.28	37.92 ± 8.78	37.51 ± 8.42	3.02 ± 0.42	3.01 ± 0.23	6.38 ± 0.85	6.50 ± 0.78
1B-JHMfeI-T Mean ± SD	30.61 ± 6.04	30.82 ± 6.72	29.71 ± 5.78	2.82 ± 0.40	2.79 ± 0.20	5.94 ± 0.74	6.07 ± 0.53
*U*-test	6.547**	6.040**	6.427**	4.988**	5.740**	5.184**	5.509**
2A-CAPSmin-T^*a*^ Mean ± SD	36.20 ± 8.18	36.01 ± 7.95	35.08 ± 8.04	3.00 ± 0.36	2.94 ± 0.22	6.28 ± 0.72	6.38 ± 0.64
2A-CAPSmin-C Mean ± SD	27.61 ± 6.24	29.01 ± 7.70	27.88 ± 6.43	2.78 ± 0.39	2.72 ± 0.22	5.95 ± 0.77	6.06 ± 0.77
*U*-test	6.256**	5.056**	4.748**	4.877**	4.659**	3.013**	2.445*
4B-8621-C^*a*^ Mean ± SD	38.40 ± 7.34	38.39 ± 7.17	37.93 ± 6.15	3.07 ± 0.35	3.02 ± 0.18	6.45 ± 0.67	6.62 ± 0.48
4B-8621-G Mean ± SD	31.57 ± 7.53	31.37 ± 8.17	30.50 ± 7.96	2.82 ± 0.43	2.82 ± 0.24	5.95 ± 0.85	6.05 ± 0.72
*U*-test	5.863**	6.039**	6.169**	5.714**	5.571**	5.588**	5.943**
7A-3738-G^*a*^ Mean ± SD	39.26 ± 7.54	39.09 ± 7.41	37.92 ± 7.63	3.09 ± 0.32	3.04 ± 0.20	6.48 ± 0.70	6.55 ± 0.73
7A-3738-C Mean ± SD	29.73 ± 5.68	29.72 ± 6.83	29.12 ± 6.03	2.76 ± 0.44	2.77 ± 0.20	5.85 ± 0.80	6.01 ± 0.54
*U*-test	8.077**	7.772**	7.365**	7.084**	7.121**	7.255**	6.735**

* and ** represent significance at 0.05 and 0.01 probability level, respectively. ^*a*^The favorable allele with Jing 411-type band and high grain weight/size.

### Candidate Gene Prediction for *Qtgw.ahau-4B.2*

The new stable and major QTL *Qtgw.ahau-4B.2* was flanked by two CAPS markers, 4B-2133 and 4B-8416, in a physical interval of 0.31 Mb (Table 1). According to IWGSC annotation databases, the target region of *Qtgw.ahau-4B.2* contains 19 genes. Among them, *TraesCS4B02G376400* and *TraesCS4B02G376800*, which encode a plasma membrane H^+^-ATPase and a serine/threonine-protein kinase, respectively, were considered as potential candidate genes (Supplementary Table 10).

### Cloning of *TraesCS4B02G376400* and Validation in 180 CMCCs

**FIGURE 2:**
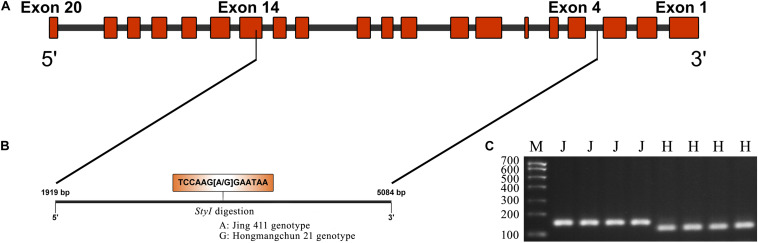
CAPS marker development based on a SNP in *TraesCS4B01G376400.* **(A)** Diagram of the gene structure of *TraesCS4B01G376400*. **(B)** SNP (A–G) found in the partial sequence (1919–5084 bp) of *TraesCS4B01G376400*. **(C)** Electrophoresis patterns of the CAPS marker PMA-2 digested by *Sty*I between two parents Jing 411 and Hongmangchun 21. M indicates marker. “J” and “H” indicate electrophoresis patterns of Jing 411 genotype (undigested) and Hongmangchun 21 genotype (digested).

Sequence analysis indicated that *TraesCS4B02G376400* comprised 20 exons and 19 introns, with a complete sequence of 6024 bp (Figure 2A). After partial sequencing of *TraesCS4B02G376400* (1919–5084 bp), a SNP (A-G) was identified in the 12th intron between Jing 411 and Hongmangchun 21 (Figure 2B). Then, a gene-specific marker, PMA-2, was developed based on this SNP and used to detect allelic variations of *TraesCS4B02G376400* among 180 CMCCs (Figure 2C). Compared with the Jing 411-type (*PMA-2-A*), the Hongmangchun 21-type (*PMA-2-G*) was significantly associated with lower TGW, GW, and GL values across environments (*P* < 0.01) (Table 3).

**TABLE 3:** Association of allelic variations of the CAPS marker PMA-2 for *Qtgw.ahau-4B.2* with TGW, GW, and GL in 180 CMCC.

**Trait**	**Allele**	**Mean ± SD**	***U-test***
2015TGW-CMCC	PMA-2-A	36.357.45	4.64**
	PMA-2-G	29.295.45	
2016TGW-CMCC	PMA-2-A	36.217.83	3.84**
	PMA-2-G	29.876.61	
2017TGW-CMCC	PMA-2-A	35.507.54	3.49**
	PMA-2-G	29.286.66	
2016GW-CMCC	PMA-2-A	6.300.73	3.34**
	PMA-2-G	5.940.72	
2016GW-CMCC	PMA-2-A	6.450.61	3.27**
	PMA-2-G	6.090.29	
2016GL-CMCC	PMA-2-A	3.000.37	3.57**
	PMA-2-G	2.760.46	
2017GL-CMCC	PMA-2-A	2.950.22	2.65**
	PMA-2-G	2.790.24	

PMA-2-A indicates the Jing 411-type associated with higher TGW, GW, and GL values, PMA-2-G indicates the Hongmangchun 21-type associated with lower TGW, GW, and GL values. **Represents significance at 0.01 probability level.

## Discussion

### Reliability of QTL Mapping

The reliability of QTL mapping is crucial for subsequent fine mapping and map-based cloning of target genes and is affected by the density and genotyping of markers used to construct genetic linkage maps (Cui et al., 2017; Han et al., 2019). Recently, RAD-seq (Baird et al., 2008), GBS-seq (Elshire et al., 2011), SLAF-seq (Sun et al., 2013), and SNP arrays (Wang et al., 2014)^10^ were performed as low-cost and efficient strategies to develop abundant SNPs to increase the density of markers. In the present study, SLAF tags (4820), CAPS (74), and gene-specific markers (11) were collectively used to build a high-density genetic linkage map harboring 1614 bin markers spanning 2014.43 cM in length with an average marker interval of 1.22 cM (Supplementary Figure 1).

It is well known that different mapping methods are based on specific genetic models as well as their corresponding statistical hypothesis, from which the result is in fact a probability statement. Therefore, it is better to use multiple mapping methods to reduce the risks of identifying ghost QTLs or missing real QTLs (Su et al., 2010). Zhang et al. (2016) identified 63 and 16 additive QTLs for fiber strength of cotton using a composite interval mapping (CIM) method with WinQTLCartographer2.5 and an ICIM model by QTL IciMapping4.1, respectively, and four QTLs detected by both CIM and ICIM were considered as stable loci for the fiber strength of cotton. In our previous study, based on both single- and multi-locus MLMs, 23 and 39 MTAs for preharvest sprouting resistance were detected by GWAS, respectively, and 6 loci were jointly identified by the two models and were, thus, considered stable loci (Zhu et al., 2019).

In addition, a combination of QTL analysis and GWAS is also an important strategy to detect reliable loci. Wang et al. (2018) identified 41 QTLs for maize tocopherol content by linkage mapping in 6 RIL populations and 32 significant loci by GWAS in a diverse panel of 508 inbred lines, and a major QTL co-localized in both linkage analysis and GWAS was finely mapped and characterized as a non-tocopherol pathway gene involved in the modulation of natural tocopherol variation.

In this study, QTL analysis with three mapping methods (ICIM, GCIM, and NWIM) and GWAS with MLM were collectively performed to identify loci for grain weight and size. Notably, five QTLs for TGW, two for GW, and four for GL were identified by all three mapping methods in JH-RILs. Six of them, including *Qtgw.ahau-4B.1*, *Qtgw.ahau-4B.2*, *Qgw.ahau-2D*, *Qgw.ahau-4B.3*, *Qgl.ahau-2A.1*, and *Qgl.ahau-7A.2*, were validated by GWAS in 192 WVs. Thereby, these loci were considered to be reliable (Table 1, Supplementary Table 9, and Supplementary Figure 2).

### Comparisons With Previous Studies

All QTLs for grain weight and size detected in the present study were compared with previously reported loci based on the wheat reference genome sequence (IWGSC RefSeq 1.0; IWGSC, 2018). In total, 23 (76.7%) of 30 QTLs for TGW, 11 (73.3%) of 15 for GW, and 13 (72.2%) of 18 for GL were completely or partially coincident with previous results (Supplementary Table 8). *Qtgw.ahau-2D* and *Qgw.ahau-2D.3* (543–561 Mb) were detected close to a major QTL for TGW (*Q.TKW.ui-2D-1*, which is flanked by cfd73 and IWB1093, 553 Mb) that was identified by Liu et al. (2018). *Qgl.ahau-4B.1* (31–38 Mb), identified in JH-RILs, was close to a major and stable locus for TGW (TKW-AX_110713957, 42 Mb) (Li et al., 2019). Moreover, *Qtgw.ahau-6B.1* and *Qgl.ahau-6B* (676–679 Mb) were detected near a stable QTL for TGW (TKW-AX_109917592, 675–677 Mb) that was identified by Li et al. (2019) (Table 1).

It is noteworthy that six QTL regions detected in this study contained eight reported genes for grain weight and size, including *TaFlo2-A1* (2A) (Sajjad et al., 2017), *TaSnRK2.9-5A* (Rehman et al., 2019), *TaGL3-5A* (Yang et al., 2019a), *TaGW2-6A* (Su et al., 2012; Yang et al., 2012; Hong et al., 2014; Qin et al., 2014; Vandana et al., 2015; Wang et al., 2017), *TaSus1-7A* (Hou et al., 2014), *TaGASR7-7A* (Dong et al., 2014), and *TaTGW-7A* (Hu et al., 2016b). This supports the reliability of QTL mapping performed in the current study. However, most of these reported genes had minor effects on grain weight and size traits in JH-RILs except for *Qtgw.ahau-7A.1* (co-located with *Qgw.ahau-7A.1* and *Qgl.ahau-7A.1*), indicating that control of grain weight and size is multigenic (Supplementary Table 8).

In particular, several putative, novel QTLs were identified in the present study compared to previous studies. For instance, *Qtgw.ahau-4B.1*, *Qtgw.ahau-4B.2*, and *Qgl.ahau-2A.2*, which explained 12.4, 11.7, and 12.1% of the phenotypic variances, on average, respectively, have not been reported by previous studies and are, thus, regarded as novel loci (Table 1 and Supplementary Table 8). Interestingly, two loci for TGW and GL detected by all three mapping methods (*Qtgw.ahau-1B.1* and *Qgl.ahau-7A.2*) in the present study overlapped with the reported QTLs associated with kernel number per spike (KNS) (Cui et al., 2014; Wang et al., 2017), indicating a pleiotropy or close linkage between them (Table 1 and Supplementary Table 8).

### Pleiotropic or Co-localized QTLs

Thousand grain weight shows significant, positive correlations with GW and GL as shown previously and in the current study (Sun et al., 2009; Drikvand et al., 2013; Wang J. et al., 2020) (Supplementary Tables 5, 6), suggesting that grain weight and size were simultaneously selected in high-yield wheat breeding. In this study, several QTL intervals were identified for multiple yield-related traits. For example, two pairs of QTLs related to TGW and GW were detected in same intervals on chromosomes 2D and 4A; four QTL intervals related to both TGW and GL were identified on chromosomes 1B, 2A, 3A, and 6B; two QTL intervals for both GW and GL were detected on chromosomes 2D and 4B. Moreover, six QTL intervals associated with TGW, GW, and GL were identified on chromosomes 1B, 4B (2), 7A (2), and 7B, respectively. These include four novel QTL intervals, 648–652 Mb of chromosome 1B (*Qtgw.ahau-1B.1*, *Qgw.ahau-1B.1*, and *Qgl.ahau-1B.1*), 540–542 Mb of chromosome 4B (*Qtgw.ahau-4B.1*, *Qgw.ahau-4B.2*, and *Qgl.ahau-4B.2*), 660.11–660.42 Mb of chromosome 4B (*Qtgw.ahau-4B.2*, *Qgw.ahau-4B.3*, and *Qgl.ahau-4B.4*), and 446–496 Mb of chromosome 7A (*Qtgw.ahau-7A.2*, *Qgw.ahau-7A.2*, and *Qgl.ahau-7A.2*). Interestingly, four novel QTL intervals for grain weight and size identified in this study were co-located with QTLs for KNS detected in previous research, including *Qtgw.ahau-1B.1* (co-located with *Qgl.ahau-1B.1*) (Cui et al., 2014), *Qtgw.ahau-3A.2* (Li et al., 2019), *Qtgw.ahau-4D* (Cui et al., 2014), and *Qtgw.ahau-7A.2* (co-located with *Qgw.ahau-7A.2* and *Qgl.ahau-7A.2*) (Wang et al., 2017). In brief, these findings indicate that the above QTL intervals are either pleiotropic or tightly linked regions controlling multiple yield-related traits.

### Candidate Gene Prediction of *Qtgw.ahau-4B.2*

The new stable locus *Qtgw.ahau-4B.2* spanning a physical interval of 0.31 Mb was detected in 10 of 11 environments in which 19 genes were annotated (Table S10). Among them, *TraesCS4B02G376400* and *TraesCS4B02G376800* encode a plasma membrane H^+^-ATPase and a serine/threonine-protein kinase, respectively. The plasma membrane (PM) H^+^-ATPase is an important ion pump in the plant cell membrane. By extruding protons from the cell and generating a membrane potential, this pump energizes the PM, which is a prerequisite for plant growth. Thus, the PM H^+^-ATPase is regarded as a driver of growth (Falhof et al., 2016). Rober-Kleber et al. (2003) indicated that the PM H^+^-ATPase is involved in auxin-mediated cell elongation during wheat embryo development. Auxin activates the proton pump, resulting in apoplastic acidification that contributes to cell wall loosening and elongation of the scutellum. Therefore, the PM H^+^-ATPase is a component of the auxin-signaling cascade that may direct pattern formation in embryos. Moreover, several reported genes related to TGW belong to the serine/threonine-protein kinase family, such as *TaSnRK2.3* (Miao et al., 2017), *TaSnRK2.9* (Rehman et al., 2019), *TaSnRK2.10* (Zhang Z. et al., 2017), and *Tasg-D1* (Cheng et al., 2020), implying that the serine/threonine-protein kinase proteins play important roles in regulation of wheat grain development. Taken together, *TraesCS4B02G376400* and *TraesCS4B02G376800* are likely to be candidate genes of *Qtgw.ahau-4B.2*.

## Conclusion

A total of 30, 15, and 18 putative additive QTLs for TGW, GW, and GL, respectively, were identified by SLAF-map in JH-RILs using three mapping methods. Particularly, five novel QTLs with stable and significant effects, including *Qtgw.ahau-1B.1*, *Qgl.ahau-2A.1*, *Qtgw.ahau-4B.1*, *Qtgw.ahau-4B.2*, and *Qgl.ahau-7A.2* were identified by all three mapping methods and further validated in a natural population. In addition, *TraesCS4B02G376400* and *TraesCS4B02G376800* were considered as potential candidate genes underlying *Qtgw.ahau-4B.2*. The novel QTLs and CAPS markers developed will be helpful for map-based cloning of the target regions and gene pyramiding in breeding for wheat PHS resistance.

## Data Availability Statement

The datasets presented in this study can be found in online repositories. The names of the repository/repositories and accession number(s) can be found in the article/Supplementary Material.

## Author Contributions

JC and YS conceived the study, put into effect the main linkage analyses, and drafted the manuscript. DX, KX, and XC took part in the experiments and drafting of the manuscript. XP, XL, ML, CG, SY, HY, and WG processed the experimental data and helped to draft the manuscript. JL, HZ, CC, XX, SX, and CM conceived and guided the experiments, and helped in coordinating the project and drafting the manuscript. All the authors read and accepted the final manuscript.

## Conflict of Interest

The authors declare that the research was conducted in the absence of any commercial or financial relationships that could be construed as a potential conflict of interest. The handling Editor declared a past collaboration with one of the authors SX.

## Footnotes

1 https://urgi.versailles.inra.fr/blast_iwgsc/blast.php

2 https://gatk.broadinstitute.org

3 http://www.illumina.com

4 https://www.PremierBiosoft.com

5 https://www.neb-china.com

6 http://www.isbreeding.net

7 https://cran.r-project.org/web/packages/QTL.gCIMapping.GUI/index.html

8 https://wheat-urgi.versailles.inra.fr/Tools/JBrowse

9 http://www.lynnon.com/

10 https://www.cerealsdb.uk.net/cerealgenomics/CerealsDB/indexNEW.php

## Supplementary Material

Supplementary Figure 1 Genetic map containing 1614 bin markers for linkage mapping in the Jing 411/Hongmangchun 21 RILs population.

Supplementary Figure 2 Manhattan plots and Quantile–quantile (Q-Q) plots for TGW, GW, and GL of 192 WVs (lines) by the MLM in Tassel v5.0.

Supplementary Figure 3 Electrophoresis patterns of four CAPS markers 1B-JHMfeI, 2A-CAPSmin, 4B-8621 and 7A-3738 between two parents Jing 411 and Hongmangchun 21.

Supplementary Table 1 Information of 74 CAPS markers developed in this study.

Supplementary Table 2 Information on 11 gene-specific markers.

Supplementary Table 3 Primers used for cloning and marker development of *TraesCS4B02G376400.*

Supplementary Table 4 Phenotypic data of grain weight and size in the Jing 411/Hongmangchun 21 RIL population and 192 WVs.

Supplementary Table 5 Pearson’s correlations between TGW, GW, and GL in the Jing 411/Hongmangchun 21 RIL population.

Supplementary Table 6 Pearson’s correlations between TGW, GW, and GL in 192 WVs.

Supplementary Table 7 Summary of the high-density genetic linkage bin maps constructed by SLAF-seq, CAPS, and gene-specific markers in the Jing 411/Hongmangchun 21 population.

Supplementary Table 8 QTLs for grain weight and size identified in the Jing 411/Hongmangchun 21 RIL population.

Supplementary Table 9 Thirty-one markers for grain weight and size in 192 WVs identified by GWAS.

Supplementary Table 10 Gene annotation of the *Qtgw.ahau-4B.2* interval.
