# Supplementary figures and images for: Identification and Validation of New Stable QTLs for Grain Weight and Size by Multiple Mapping Models in Common Wheat
Source: Front Genet. 2020 Nov 11;11:584859. doi: 10.3389/fgene.2020.584859 (PMC7686802; doi:10.3389/fgene.2020.584859)

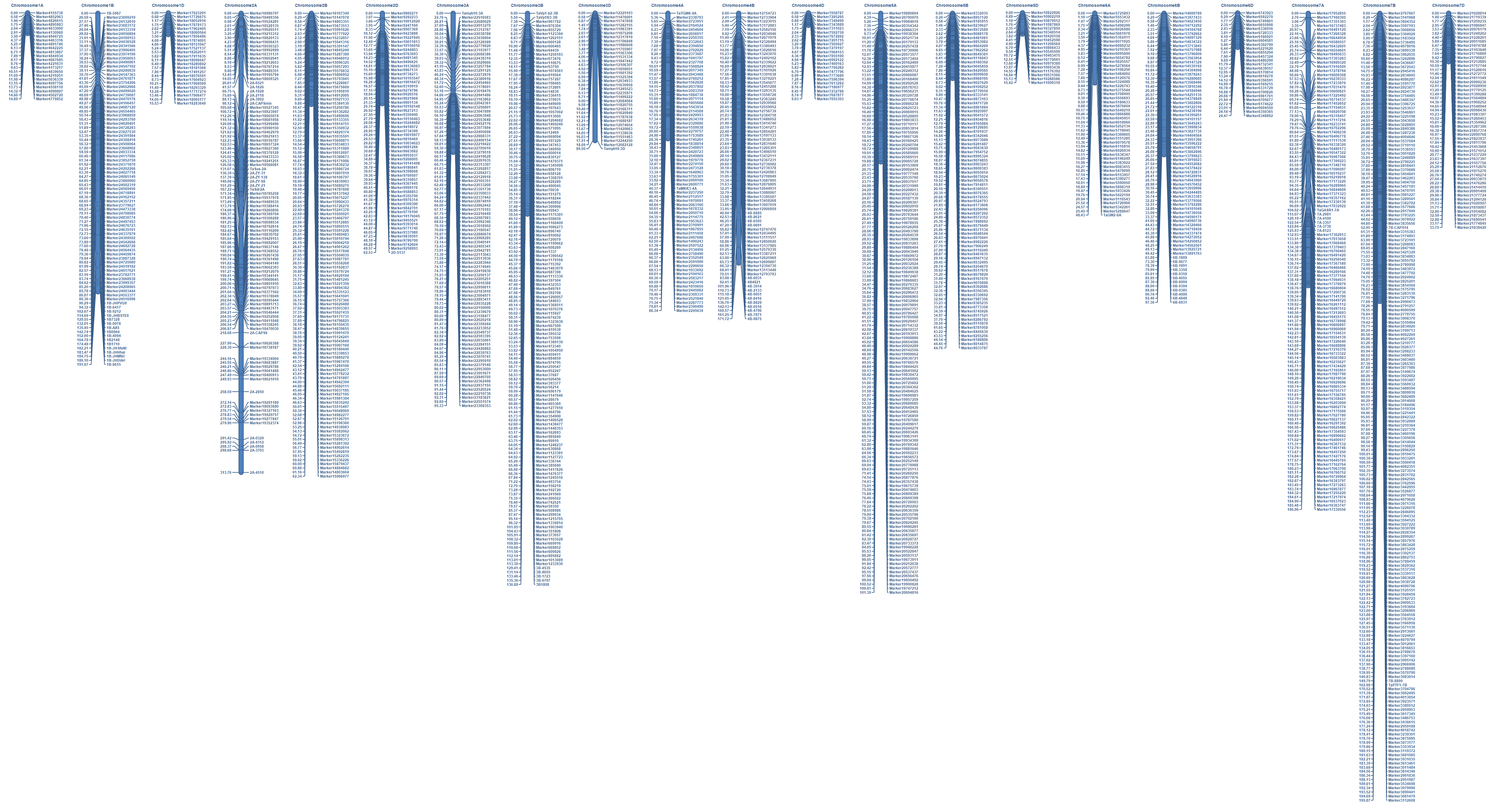

Supplement: Supplementary Figure 1 — Genetic map containing 1614 bin markers for linkage mapping in the Jing 411/Hongmangchun 21 RILs population. [file Image_1.TIF]

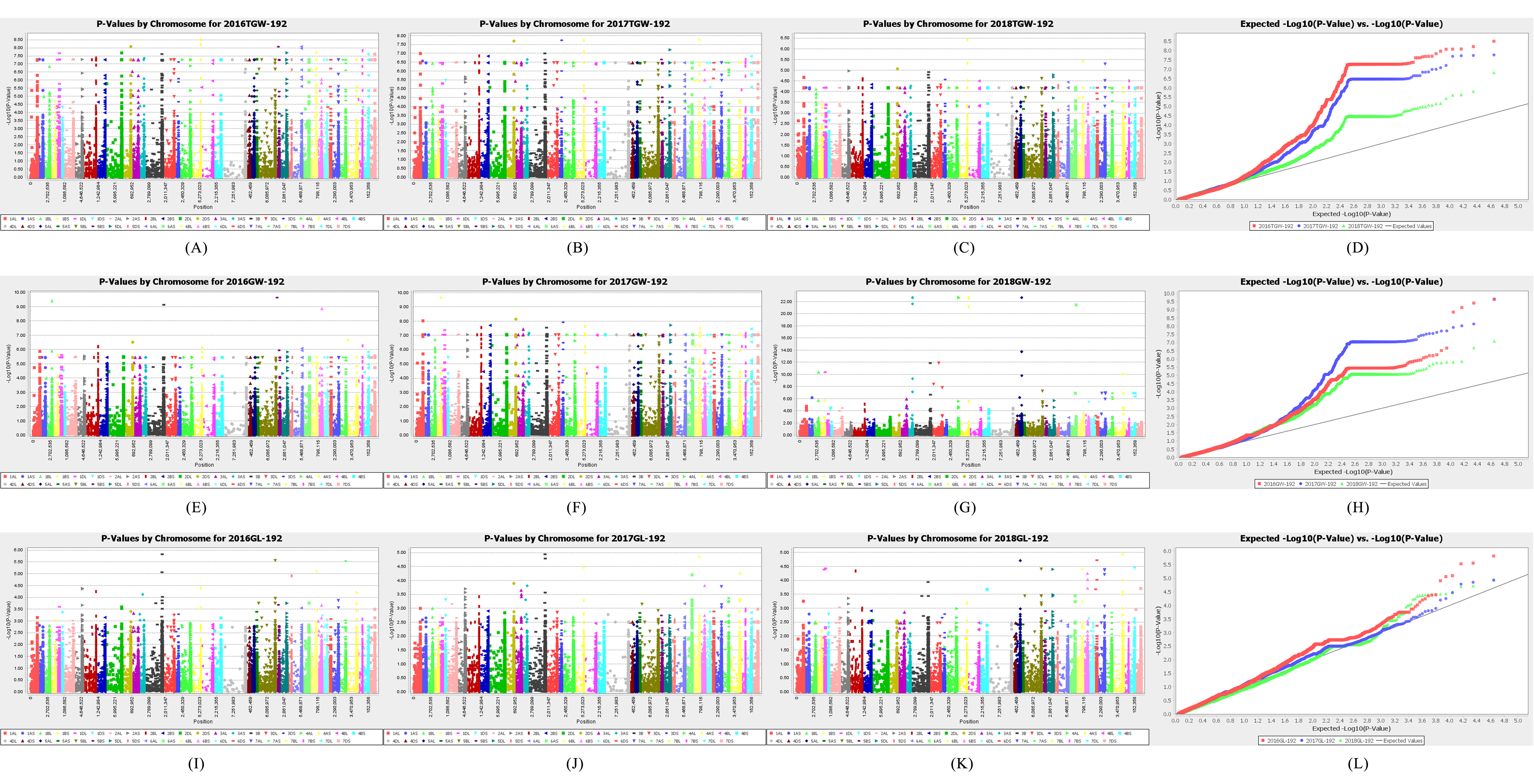

Supplement: Supplementary Figure 2 — Manhattan plots and Quantile–quantile (Q-Q) plots for TGW, GW, and GL of 192 WVs (lines) by the MLM in Tassel v5.0. [file Image_2.TIF]

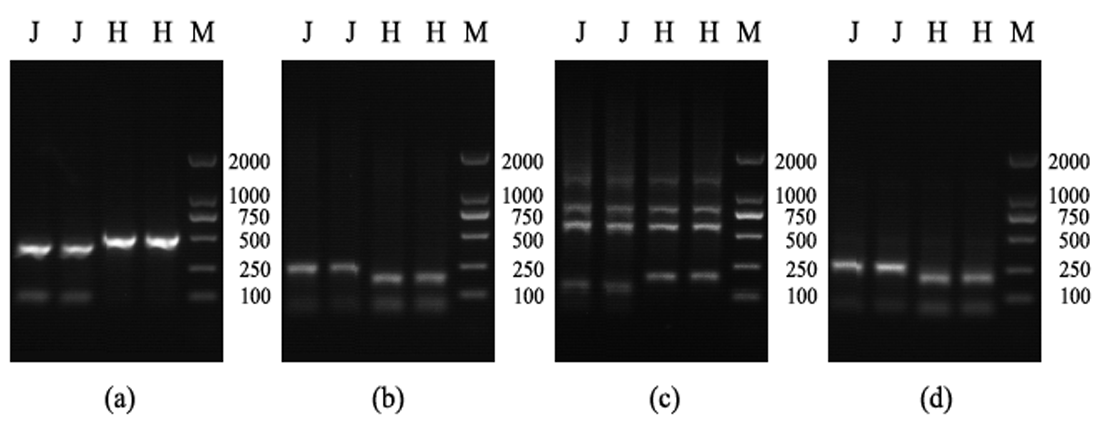

Supplement: Supplementary Figure 3 — Electrophoresis patterns of four CAPS markers 1B-JHMfeI, 2A-CAPSmin, 4B-8621 and 7A-3738 between two parents Jing 411 and Hongmangchun 21. [file Image_3.TIF]
